# A pH-Responsive Poly Beta-Amino Ester Nanoparticulate Thermo-Responsive PEG-PCL-PEG Hydrogel Dispersed System for the Delivery of Interferon Alpha to the Ocular Surface

**DOI:** 10.3390/pharmaceutics17060709

**Published:** 2025-05-28

**Authors:** Yosra Abdalla, Lisa Claire du Toit, Philemon Ubanako, Yahya Essop Choonara

**Affiliations:** 1 Wits Advanced Drug Delivery Platform Research Unit, Department of Pharmacy and Pharmacology, School of Therapeutic Sciences, Faculty of Health Sciences, University of the Witwatersrand, Parktown, 7 York Road, Johannesburg 2193, South Africalisa.dutoit1@wits.ac.za (L.C.d.T.); 2 Wits Infection Diseases and Oncology Research Institute (IDORI), Faculty of Health Sciences, University of the Witwatersrand, Parktown, 7 York Road, Johannesburg 2193, South Africa; 3 Department of Internal Medicine, School of Clinical Medicine, Faculty of Health Sciences, University of the Witwatersrand, Parktown, 7 York Road, Johannesburg 2193, South Africa; philemon.ubanako@wits.ac.za

**Keywords:** ocular tumours, interferon alpha, pH-responsive/nanoparticles, thermo-responsive hydrogel, acidic tumour microenvironment

## Abstract

**Background/Objectives:** The management of ocular tumours is faced with the challenge of developing a suitable treatment strategy with consideration of the anatomical and physiological protective barriers of the eye. Interferon alpha has been employed to treat patients with ocular tumours for decades; however, its short half-life and poor tolerability necessitate frequent administration. This study focuses on the design of an injectable pH-responsive and protective nanoparticle system dispersed into a thermo-responsive hydrogel for site-specific sustained delivery of interferon alpha (IFN-α2b) in the treatment of ocular surface tumours. **Methods:** The synthesis of a poly(ethylene glycol)-poly(caprolactone)-poly(ethylene glycol) (PEG-PCL-PEG) triblock copolymer (PECE) was undertaken. The IFN-α2b was encapsulated in poly(β-amino ester) (PBAE) nanoparticles (NP) with pH-responsive characteristics to proposedly release the IFNα-2b in response to the acidic nature of the tumour microenvironment. This was followed by characterisation via Fourier transform infrared spectroscopy (FT-IR), ^1^H-nuclear magnetic resonance (^1^H-NMR) analysis, differential scanning calorimetry (DSC), X-ray powder diffraction (XRPD) analysis, thermogravimetric analysis (TGA), and thermal-transition analysis of the PECE hydrogels. **Results:** Release studies demonstrated that the PBAE nanoparticulate PEG-PCL-PEG hydrogel was both pH-responsive, while providing controlled release of IFN-α2b, and thermo-responsive. Release analysis highlighted that IFN-α2b-loaded NP dispersed into the hydrogel (IFNH) further prolonged the release of IFN-α2b with a pH-responsive yet controlled release rate in an acidic environment simulating a tumour microenvironment. The developed system proved to be biocompatible with human retinal pigment epithelial cells and the released IFN-α demonstrated bioactivity in the presence of an A172 glioblastoma cell line. **Conclusions:** In conclusion, the PECE hydrogel has promising potential for application as an ocular drug delivery system for the treatment of ocular tumours and could potentially overcome and prevent the drawbacks associated with the commercially available IFN-α2b injection.

## 1. Introduction

The management of ocular tumours is mainly through surgery, cryotherapy, radiotherapy, chemotherapy, and immunotherapy [1]. The first line treatment involves surgical excision employing a no-touch technique with adjuvant cryotherapy [2]. However, these strategies are fraught with challenges such as high recurrence rate and vision-threatening side effects (limbal stem cells deficiency, corneal oedema, etc.) [3]. Additionally, topically applied chemotherapy, which is usually presented as eye drops, is faced with decreased patient compliance, due to the dosing regimen. As a result, immunotherapy using IFN-α2b, is important in the management of ocular surface tumours. IFN-α2b is a cytokine produced in the body with antiviral, antiproliferative, antitumour, and immunomodulatory activities and has been used in management of other types of cancer including malignant melanoma, hairy cell leukaemia, follicular non-Hodgkin’s lymphoma and AIDS-related sarcoma [4,5]. The antitumour effect of IFN-α2b is mediated by the Janus kinases (JAK-STAT) and signal transduction pathways. Cancer cell growth suppression occurs via cell cycle arrest and apoptosis is regulated by this mechanism. Furthermore, IFN-α2b operates by inhibiting angiogenesis, activating immune cells such as T cells and natural killer cells, and inducing cytokines [6]. Application of IFNα-2b is through doses of 1–3 million IU/mL as subconjunctival/perilesional injection/s applied at single or multiple sites, or via topical application, which is applied up to four times a day [7,8]. Subconjunctival injections can be safely administered with relative ease and without the need for sophisticated equipment in the clinical setting. However, systemic flu-like symptoms including mild fever and myalgia are common in most patients but are considered mild and well tolerated [8]. Immunotherapy with IFNα-2b is hindered by its macromolecule size, hydrophilicity, rapid clearance, and degradation at low pH in the tumour microenvironment. Rapid clearance necessitates the administration of high doses, resulting in unwanted complications [9,10].

Multiple approaches have been investigated for the delivery of interferons, including coupling of proteins or polymers (e.g., PEG), nanoparticles, microspheres, and hydrogels [11,12,13]. Pegylated interferon (PEGIFN-α2b) has been investigated as an approach to limit the rapid clearance of IFN-α2b by the reticuloendothelial system (RES) and reduce immunogenicity [14]. Unfortunately, this is very expensive and complex; therefore, there remains a need for a simple approach for site-specific and sustained delivery of immunotherapy for the management of ocular malignancies.

The design of a delivery system for immunotherapeutic agents can incorporate specific polymers or ligands to target specialised characteristics displayed by the tumour microenvironment and/or cells of interest for directed delivery of therapeutic agents [15]. These characteristics include enhanced permeation and retention (EPR) effect, the acidic pH of tumour cells, and overexpressed receptors, which are identified on the surface of tumours and known as the “hallmark” of tumours [16]. The acidic extracellular pH of a tumour is an important characteristic that can be employed for controlled delivery; the pH is within a range of 6.0–7.0, whereas the extracellular pH for normal cells is at 7.4 [17]. These unique features render it possible to design a platform that protects the bioactive at normal pH and releases it in a sustained manner at the tumour sites.

PECE is an amphiphilic copolymer, with poly caprolactone (PCL) as the hydrophobic block and polyethylene glycol as the hydrophilic block. Both PCL and PEG are FDA-approved polymers that have been employed in several formulations due to their biodegradability and biocompatibility [18]. Poly (beta amino esters) (PBAE) are a class of copolymers that are cationic in nature, therefore considered appropriate for intracellular delivery of therapeutic genes, proteins, peptides, and anticancer drugs. They offer several advantageous characteristics including low toxicity, biodegradability, and efficient cell uptake and facilitate the escape of cargo from the endolysosomal system owing to their high buffer capacity [19]. The highly positive charge of PBAEs leads to rapid hydrolysis in physiological conditions rendering the NPs unstable in blood [20]. PBAEs are synthesised through Michael addition polymerisation of diacrylate esters and primary amines monomer. Modification of the polymers through conjugation with poly(ethylene glycol) or surface modification with polyelectrolyte can minimise the poor stability and enhance functionality. Previous reports have concluded that surface-modification of PBAE-NPs with HA lead to an enhanced cytotoxicity effect of DOX and prolonged in vivo circulation and stability of the NPs [20]. Gupta and co-workers reported the conjugation of PBAEs with mPEGA, the stability and release were enhanced by the applied modification of the PBAEs polymer [21].

Aiming to overcome the drawbacks of frequent subconjunctival or perilesional injection of IFN-α2b, the present research proposes the preparation of pH-responsive NPs dispersed in a thermo-responsive in situ hydrogel, designed for site-specific ocular delivery of IFN-α2b. The IFN-α2b was encapsulated into pH-responsive poly(beta amino ester) nanoparticles (PBAE-NPs) and suspended in a poly(ethylene glycol)-poly(caprolactone)-poly(ethylene glycol) hydrogel PECE. The nanoparticle-embedded PECE hydrogel matrix with its core-shell structure is expected to enclose the bioactive, protecting it from the cursive environment and prolonging the release pathway, while promoting IFN-α2b release at tumour extracellular microenvironment pH versus normal physiological pH. The system is proposed to display both pH- and thermo-responsive behaviour, gelling following injection into the subconjunctival space of the eye, and releasing IFNα-2b in a pH-responsive and sustained manner at the tumour site.

## 2. Materials and Methods

### 2.1. Materials

1,4-Butanediol diacrylate (BDDA, Mw = 198.22), poly(ethylene glycol) methyl ether acrylate (mPEGA, Mw = 480), 3-Amino-1-propanol (3AP, Mw = 75.11), poly(ethylene glycol) methyl ether (mPEG, Mn = 550), Ɛ-caprolactone (Ɛ-CL, Mw = 114.14), stannous octoate (Sn(Oct)_2_), and 1,6-hexamethylene diisocyanate (HMDI) were purchased from Sigma Aldrich (St. Louis, MO, USA). Human retinal pigment epithelial cells (HRPE) were obtained from ATCC (Manassas, VA, USA), 3-(4, 5-dimethylthiazol-2-yl)-2,5-diphenyl tetrazolium bromide (MTT) was purchased from Sigma Aldrich (St. Louis, MO, USA). Dulbecco’s modified Eagle medium mixture with Ham’s (DMEM F12), Rhodamine phalloidin (RP), DAPI stain, and enzyme-linked immunosorbent assay kit (ELISA) catalogue no BMS216 were purchased from Thermofisher (Vienna, Austria). All other reagents and solvents used were of analytical grade and used as received.

### 2.2. Methods

#### 2.2.1. Synthesis and Purification of PEG-PCL-PEG Triblock Copolymer

**Figure 1:**
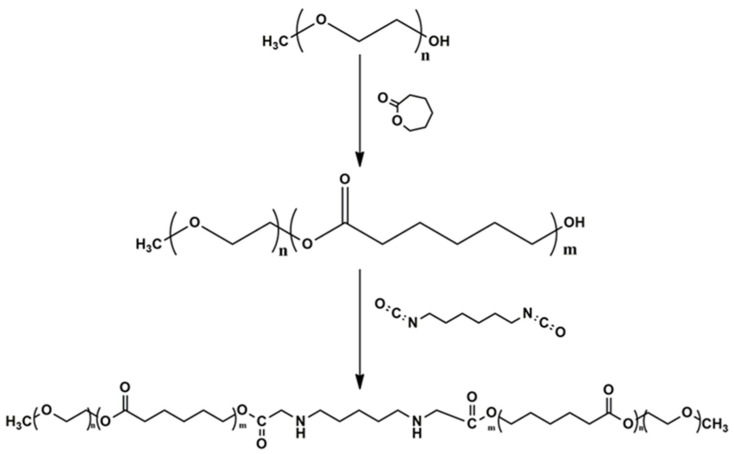
Synthetic scheme of PEG-PCL-PEG triblock copolymer.

The di-block polymer PEG-PCL was synthesised through ring opening polymerisation of Ɛ-CL using mPEG as a macro-initiator and stannous octoate Sn(Oct)_2_ as a catalyst, while the triblock copolymer PEG-PCL-PEG was synthesised by using HMDI as a coupling agent as shown in Figure 1, as previously described by Gong et al. (2009) [18]. Briefly, 10.67 mL (0.01 mol) of Ɛ-CL, 5 mL (0.01 mol) of mPEG and 0.5% *w*/*w* of Sn(Oct)_2_ were transferred, under nitrogen, to a three-necked round bottom flask mounted on a magnetic stirrer. The reaction was allowed to proceed at 130 °C for 12 h. Thereafter, the HMDI was added to the reaction flask at 80 °C and the reaction continued for 6 h. The resulting copolymer mixture was cooled to room temperature, dissolved in dichloromethane (DCM), and subsequently precipitated using petroleum ether. The precipitate was dried under vacuum at room temperature to a constant weight [22].

#### 2.2.2. Synthesis of Poly (Beta-Amino Ester) Copolymer

**Figure 2:**
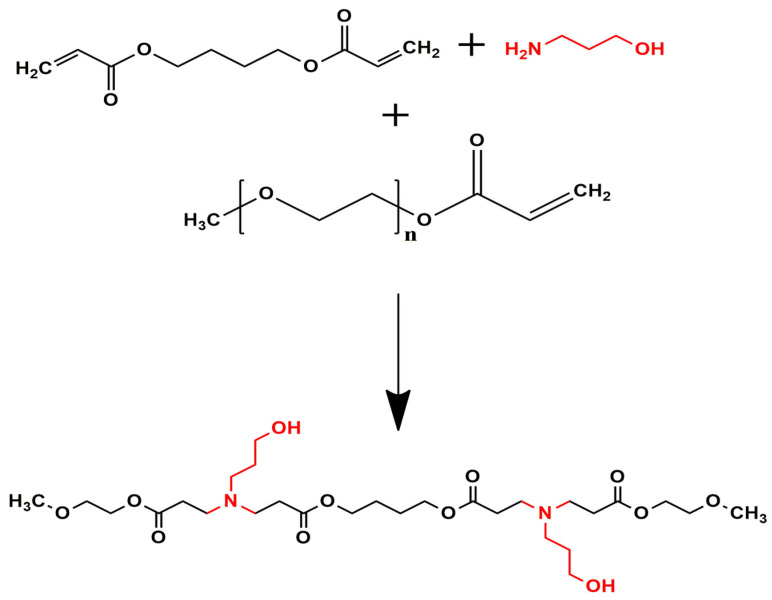
Synthetic scheme of the PBAE copolymer.

The poly(beta-amino ester) (PBAE) copolymer was synthesised via Michael addition polymerisation as shown in Figure 2, from predetermined amounts of 3AP, BDDA, and mPEGA [23,24]. Briefly, at a (1:1.1) ratio of 3AP to BDDA were transferred into glass vials containing 5 mL of tetrahydrofuran (THF) to dissolve the mixture. Thereafter, the mixture was transferred to a three-neck round bottom flask containing a solution of mPEGA in 5 mL THF. The reaction flask was mounted on a magnetic stirrer, under nitrogen; the reaction was allowed to proceed at 50 °C for 48 h. The resulting copolymer mixture was cooled to room temperature, precipitated using a large excess of cold diethyl ether, and dried under vacuum at room temperature to a constant weight.

#### 2.2.3. Preparation of Nanoparticles via the Solvent Evaporation Method

Poly(beta-amino ester) nanoparticles (PBAE-NPs) were prepared using the solvent evaporation approach [25]. Briefly, 20 mg of the PBAE copolymer was dissolved in 2 mL of acetone. IFN-α2b aqueous solution (200 µL; 1 ng/mL) was added to the organic phase, and the two phases stirred for 30 s. This mixture was added dropwise to 5 mL 1% *w*/*w* aqueous Span^®^ 20 solution and stirred at 500 rpm for 3 h to evaporate the acetone. The dispersed IFN-α2b NPs were stored at −20 °C for further analysis. The same procedure applied for the preparation of the unloaded PBAE-NPs.

#### 2.2.4. Preparation of the PECE Hydrogel and NP-Loaded/PECE Hydrogel

The PECE hydrogel was prepared by dissolving the required amount of PECE in deionized water. Thereafter, the mixture was heated in a water bath at 55 °C for 2 min and cooled, under stirring, in an ice bath to 0 °C for 10 min. The NP-loaded PECE hydrogels NP/PECE were prepared by adding the required amount of the NPs into the PECE solution under stirring. The percentage of the NPs in the NP/PECE composite was expressed as the mass ratio of the NPs (W_NPs_) to the total mass of the solid component (W_NPs_ + W_PECE_). The interferon-alpha2b nano-embedded PECE hydrogel (IFNPH) were prepared by adding the IFN-α2b loaded NPs to the PECE hydrogel solution with stirring. The composition of the PECE hydrogels was as follows: PECE-1 (20% *w*/*v* hydrogel); PECE-2 (NP loaded into 20% *w*/*v* hydrogel); PECE-3 (25% *w*/*v* hydrogel); and PECE-4 (NPs loaded into 25% *w*/*v* hydrogel).

#### 2.2.5. Fourier Transform Infrared Spectroscopy (FTIR) of the PECE Hydrogel and NP-Loaded/PECE Hydrogel

The functional groups appearing as a result of molecular transitions in the structures of the copolymers and their starting materials were recorded using Fourier transform infrared (FTIR) spectroscopy using a spectrophotometer (PerkinElmer Spectrum 100, Beaconsfield, UK). FTIR spectra scans were obtained in the region of 4000–650 cm^−1^ with a resolution of 4 cm^−1^.

#### 2.2.6. H-Nuclear Magnetic Resonance Analysis of the PECE Hydrogel and NP-Loaded/PECE Hydrogel

**^1^**H-nuclear magnetic resonance (^1^H-NMR) analysis was undertaken using a 400 MHz spectrometer (300, Bruker, Fällanden, Switzerland) and deuterated chloroform (CDCl_3_) as a solvent to confirm the synthesis of the copolymers.

#### 2.2.7. X-Ray Powder Diffraction (XRPD) Analysis of the PECE Hydrogel and NP-Loaded/PECE Hydrogel

The degree of crystallinity of the lyophilized PECE copolymer, PECE-1 and PECE-2 were analysed using XRPD spectra (Rigaku MiniFlex 600, Tokyo, Japan). The 2θ scan from 30 to 90 °C was selected at a scanning rate of 10°/min to determine the diffractogram of the samples.

#### 2.2.8. Differential Scanning Calorimetry (DSC) of the PECE Hydrogel and NP-Loaded/PECE Hydrogel

The thermal behaviour of PECE copolymer, PECE-1 and PECE-2 (refers to 25% PECE hydrogel and PBAE-NPs/25% PECE, respectively) were analysed employing a differential scanning calorimeter (DSC) (Mettler Toledo, Greifensee, Switzerland) equipped with STARe SW software (version V18.00b). The lyophilised samples were weighed and placed into aluminium crucibles. The samples were subjected to a heating cycle from 0 to 70 °C and cooled from 70 to 0 °C with a heating rate of 3 °C/min under the flow of nitrogen.

#### 2.2.9. Thermogravimetric (TGA) Analysis of the PECE Hydrogel and NP-Loaded/PECE Hydrogel

The thermal degradation of PECE copolymer, PECE-1 and PECE-2 was analysed using a thermogravimetric analyser (TGA) (PerkinElmer, TGA 4000, Llantrisant, Wales, UK). The lyophilized samples were subjected to a heating gradient from 30 to 900 °C with a heating rate of 10 °C/min under the flow of nitrogen.

#### 2.2.10. Particle Size Distribution, Polydispersity Index (PDI), and Zeta Potential Analysis of the Nanoparticles

The dimensions of the unloaded and loaded NPs were measured by dynamic light scattering (DLS) using a Malvern ZetaSizer Nano ZS (Malvern Instruments Ltd., Malvern, UK) at 25 °C. The particle size, zeta potential and PDI were measured in disposable cuvettes. Sample measurements were conducted in triplicate, and the mean value was reported accordingly.

#### 2.2.11. Evaluation of Drug Entrapment Efficiency of IFN-α2b Loaded Nanoparticles

The encapsulation efficiency (EE) of IFN-α2b in the nanoparticles was measured indirectly. The loaded nanoparticle suspension (2 mL) was centrifuged for 25 min at 30,000× *g*. The concentration of IFN-α2b in the supernatant was measured using an ELISA kit (catalogue no BMS216), the assay was carried out in accordance with the manufacturer’s instructions. The absorbance was measured at 450 nm as the primary wavelength and 620 nm as reference wavelength using a Victor X3 plate reader (PerkinElmer, Biocompare, San Francisco, CA, USA). The procedure was conducted to determine the percentage encapsulation efficiency (% EE) via Equation (1).

(1) $$\%\;EE=\frac{Total\;quantity\;of\;IFN\alpha 2b-Quantity\;of\;free\;IFN-\alpha 2b}{Total\;quantity\;of\;IFN-\alpha 2b}\times 100$$

#### 2.2.12. Morphological Analysis of the NPs and NP-Loaded/PECE Hydrogel

##### Transmission Electron Microscopy of the Nanoparticles

High Resolution Transmission Electron Microscopy (TEM) (version TECNAIF3OST-TEM software) was employed for the imaging of the PBAE-NPs. The nanoparticle suspension was drop-cast on a carbon-coated copper grid. Samples were allowed 5 min contact time before removing the excess nanoparticles suspension using filter paper. Finally, air-drying of the samples was allowed overnight at 25 °C before observation under the microscope.

##### Scanning Electron Microscopy (SEM) of the NPs and NP-Loaded/PECE Hydrogel

The surface morphology of the prepared PBAE-NPs, PECE-1, and PECE-2 were studied using the SEM analysis (SIGMA VP, Zeiss Electron Microscopy, Carl Zeiss Microscopy Ltd.; Cambridge, UK). A drop of the samples was placed on aluminium stubs and allowed to dry. These were subsequently sputter-coated with palladium gold prior to SEM imaging.

#### 2.2.13. Porositometric and Surface Topography Analysis of the PECE Hydrogel and NP-Loaded/PECE Hydrogel

The surface area, pore size and pore volume were analysed employing the Brunauer–Emmett–Teller (BET) theory and the Barrett, Joyner and Halenda model (BJH). Lyophilized samples of PECE copolymer, PECE hydrogel and NPs/PECE were analysed using a Porositometric analyzer (Micromeritics ASAP 2020, Norcross, GA, USA). A sample weight of (0.1–0.3 g) was transferred to a sample tube and degassed. Thereafter, the sample tube was transferred to the analysis port for determination of the surface area, pore volume and pore size in accordance with the BET and BJH models. The analysis was conducted under an evacuation rate of 50 mm Hg/s with a temperature rate of 10 °C, target temperature of 40 °C and hold temperature at 30 °C for 900 min under nitrogen.

#### 2.2.14. Rheological Characterisation of the Hydrogels

The physico-mechanical properties of the PECE hydrogel as a function of temperature were evaluated using a rheometer (Haake Modular Advanced Rheometer System, Thermo Fisher Scientific, Karlsruhe, Germany). For each hydrogel sample, an appropriate volume was loaded between the two parallel plates of the Rheometer with steel cone-plate geometry of (35 mm, with gap distance of 0.052 mm). In oscillatory mode the (T**_sol-gel_**) of the formulations was measured employing the temperature ramp analysis. The experiment was performed within a range of 20 °C to 50 °C at a constant frequency of (1.0 Hz) and a heating rate of 1 °C/m. The storage modulus (G′) and the loss modulus (G″) were calculated using the RheoWin 480,00 (Haake^®^) version 4.30.0030 software. The oscillatory stress sweeps were performed at a range from (0.07 to 200 Pa) at a constant frequency of (1.0 Hz).

#### 2.2.15. Syringeability of the Hydrogels

The injectability of the hydrogels was investigated using a texture analyser (Stable Micro System TA-XT2, Surrey, UK). The tests were performed in compression mode using 21 G needles. The syringe was placed in a holder perpendicularly with the needle downward. A cylinder probe (P/50 R) was lined to the surface area of the plunger plate to extrude the hydrogel from the syringe. The test was conducted at a speed of 1 mm/s for a distance of 20 mm. The maximum force used to extrude the content out of the syringe was recorded [26,27].

#### 2.2.16. In Vitro Release Analysis of IFN-α2b from IFNPs and IFNPH

Interferon alpha release from IFN-α2b loaded PBAE-NPs (IFNPs) and IFN-α2b nano-embedded PECE hydrogel (IFNPH) was determined in phosphate buffer saline (PBS) at different pH values (6.6 and 7.4) simulating tumour pH and normal physiological conditions, respectively, with 0.02% Tween 80. The surfactant was added to prevent adsorption of IFN-α2b to the surface of the container. Briefly, the 2 mL of the IFNP suspension was transferred directly into the release medium. At specific time intervals (1, 2, 4, 8, 12 and 24 h), a 200 µL sample was withdrawn for analysis and replaced with an equivalent amount of pre-warmed release medium to maintain sink conditions. Release from IFNPH was conducted as follows: 0.5 mL of the precursor polymer solutions was transferred and pre-heated at 37 °C for a few minutes for hydrogel formation and immersed, thereafter, within the release medium. At specific time intervals (1, 3, 7, 12, up to 14 days), 200 µL samples were withdrawn for analysis and replaced with an equivalent amount of pre-warmed release medium to maintain sink conditions. The vials were placed in a thermostatically controlled orbital shaking incubator (type LM-530, Yihder Technology Co., Ltd., New Taipei, Taiwan) set at 25 rpm at 37 ± 1 °C. Samples were centrifuged at 13,000 rpm for 10 min, the supernatant was analysed for IFN-α2b release using an ELISA kit, measured at 450 nm, and reference wavelength of 620 nm using a Victor X3 plate reader (PerkinElmer, Biocompare, San Francisco, CA, USA). The quantity of IFNα-2b release was calculated from a standard linear curve of IFN-α2b in PBS (pH 7.4).

#### 2.2.17. Degradation Analysis of Formulations

Hydrogel degradation studies were performed under simulated in vitro conditions. Samples of PBAE-NP dispersed into PECE hydrogels (NPH) were prepared by weighing 6 g of each sample into vials containing 5 mL PBS pH (6.6 and 7.4), which were placed in an orbital shaking incubator maintained at 37 °C set at 50 rpm. The degree of degradation was measured at 1, 3, 7 and 14 days via measurement of the transitions in sample mass. Excess fluid was removed, and the samples were weighed and left to air dry for 72 h to ensure complete removal of moisture before obtaining the second weight to determine the eroded mass. The percentage of degradation was subsequently measured using Equation (2).

(2) $$\%\;Degradation=\frac{W_{i}-W_{t}}{W_{i}}\times 100$$

#### 2.2.18. In Vitro Cytotoxicity Analysis

The cell viability and cyto-compatibility of IFN-α2b, PBAE-NPs, PECE-1 and IFNPH in a primary human retinal pigment epithelial cell line (HRPE) was evaluated using a 3-(4,5-dimethylthiazol-2-yl)-2,5-diphenyl tetrazolium bromide (MTT) cell viability assay. The cells were maintained in culture medium DMEM/Hams F12 (1:1) supplemented with 10% foetal bovine serum albumin (FBS) and 1% penicillin-streptomycin, the culturing condition was 95% humidity, 5% CO_2_ at 37 °C. The growth medium was replaced every 48 h with fresh medium until the cells reached ~80–90% confluence. The HRPE were seeded in 96-well plates with a seeding density of 1 × 10^5^ cells/well. After 24 h, the cells were treated with PECE hydrogel, NPs, IFN-α2b and IFNPH in concentration range of (0–1000 µg/mL) in triplicates. Samples of the IFN-α2b, PBAE-NPs, PECE and IFNPH were sterilised by UV light prior to analysis. The hydrogel samples PECE-1 and IFNPH were placed in 1.5 mL centrifuge tubes covered by the culture medium and incubated in an orbital shaking incubator-horizontal (type LM-530, Yihder Technology Co., LTD.) set at 25 rpm for 7 days at 37 °C. A volume of 200 µL of the medium was sampled and used to treat the cells. Following 24 and 48 h of treating, 10 µL of MTT reagent (Merck, Darmstadt, Germany) was added, followed by 4h incubation at 37 °C. Thereafter, 100 µL of the solubilising buffer was added to dissolve the formazan crystals. The culture medium and 5% *v*/*v* DMSO served as the negative and positive controls, respectively, and the untreated wells served as the blank. The absorbance of the developed purple crystals was measured at 570 nm using a Victor X3 plate reader (PerkinElmer, Biocompare, San-Francisco, CA, USA). The percentage cell viability was subsequently measured using Equation (3).

(3) $$\%\;Cell\;viability=\frac{A_{sample}-A_{blank}}{A_{control}-A_{blank}}\times 100$$

where *A_sample_* represents the absorbance of the cells incubated with the different samples, *A_blank_* absorbance of the cells in the absence of treatment, and *A_control_* is the absorbance in the cells treated with the culture medium alone.

#### 2.2.19. Assessment of Bioactivity of the Released IFNα-2b from IFNPH

The bioactivity of the released IFNα-2b was determined by the cytotoxicity effect on a A172 glioblastoma cell line. Human cells were maintained in DMEM culture medium supplemented with 10% foetal bovine serum albumin (FBS) and 1% penicillin-streptomycin, the culturing condition was 95% humidity, 5% CO_2_ at 37 °C. The growth medium was replaced every 48 h with fresh medium until the cells reached ~80–90% confluence. The A172 were seeded in 96-well plates with a seeding density of 6 × 10^5^ cells/well. After 24 h, the cells were treated with the released IFNα-2b in triplicate. The assay was conducted under aseptic conditions, and the collection of the release samples was conducted under laminar flow. The cytotoxic effect of the released IFNα-2b was measured via an MTT assay.

#### 2.2.20. Statistical Analysis

All experiments were carried out in triplicate, and the results were expressed as the mean ± standard deviation (SD). The data were analysed using the GraphPad Prism Software (Version 5 for Windows, GraphPad Software, San Diego, CA, USA). The difference between groups was assessed using an analysis of variance (two-way ANOVA) among three groups or Student’s *t*-test between two groups. A *p*-value of ≤ 0.05 was regarded as statistically significant.

## 3. Results

### 3.1. Assessment of the Structural Modification and Integrity

**Figure 3:**
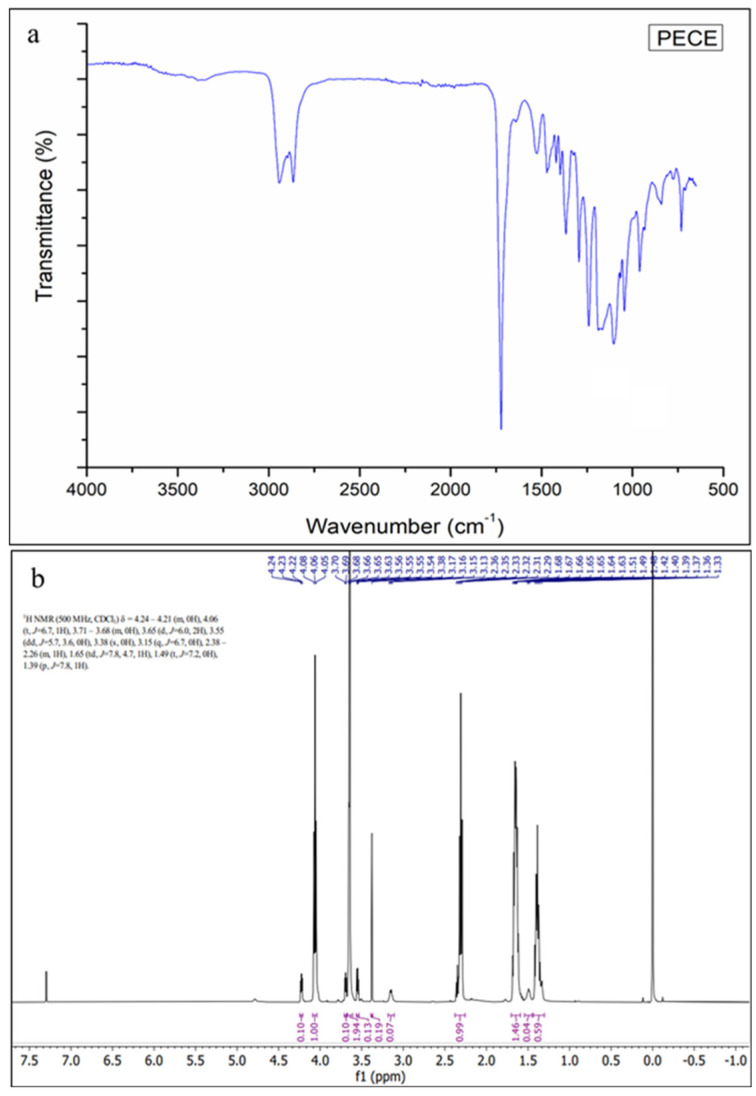
(**a**) FTIR of PECE; (**b**) H-NMR of PECE.

The chemical characteristics of the native polymers and synthesised products were investigated using FTIR. As shown in Figure 3a, the PECE triblock copolymer was characterised by an absorption peak at 1722 cm^−1^, attributed to the strong C=O stretching band of the ester bond. The absorption peaks at 2940 cm^−1^ and 2865 cm^−1^ represent the C-H stretching vibration. The absorption peak at 1527 cm^−1^ corresponds to the N-H bending vibration, confirming the formation of the PECE triblock copolymer. This is further confirmed by the disappearance of the absorption peak at 2250 cm^−1^ which corresponds to the –NCO groups of HMDI, indicating the coupling of the –OH with the –NCO group, as documented in other studies [18,22]. Further, the copolymer was characterised by ^1^H-NMR to confirm the synthesis of the PEG-PCL-PEG triblock copolymer. As evident in Figure 3b, the sharp absorption peaks at 3.6 and 3.38 ppm represent the methylene proton of CH_2_CH_2_O– and –OCH_3_ end groups in PEG blocks, respectively. The peaks at 1.39, 1.65, 2.32, and 4.06 ppm are attributed to the methylene protons of -(CH_2_)_3_, -OCCH_2_, and -CH_2_OOC- in the PCL block, respectively. The methylene protons of -O-CH_2_-CH_2_- in PEG end blocks linked with the PCL blocks appear as weak peaks at 4.2 and 3.70 ppm. The findings correlate with previous studies signifying the successful synthesis of the PECE triblock copolymer [18].

**Figure 4:**
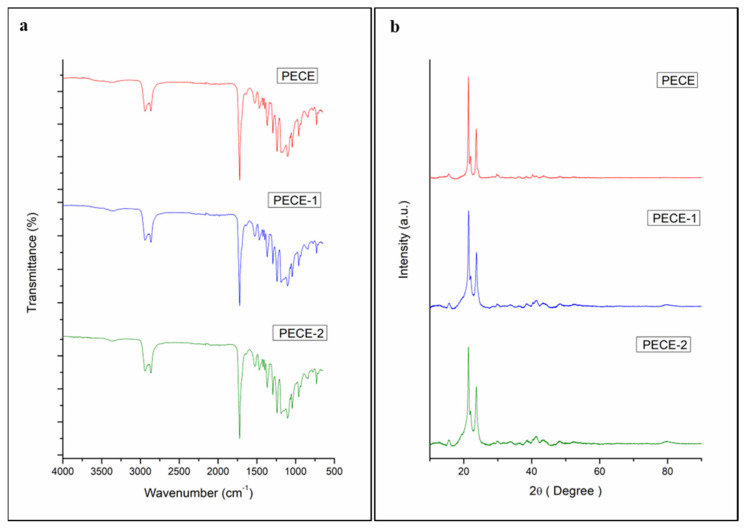
(**a**) FTIR of PECE, PECE-1 and PECE-2; (**b**) XRPD images of PECE, PECE-1 and PECE-2.

The chemical structure of the PECE copolymer and prepared hydrogels, i.e., 20% *w*/*v* PECE hydrogel (PECE-1), and nano-dispersed 20% *w*/*v* PECE hydrogel (PECE-2) showed no change in the characteristic bands indicating the change was only in the physical state of the matter from solid to hydrogel. From Figure 4a, the same characteristics peaks were detected in the hydrogel and NP-loaded hydrogel. Additionally, the crystal characteristics of the samples were evaluated using XRPD technique. This analysis was carried out for all samples (PECE copolymer, PECE-1, and PECE-2). As shown in Figure 4b, the XRPD was analysed to ascertain the crystalline structure of the PECE copolymer. Two main diffraction peaks at ~2Theta 21° and 23°, were identified corresponding to PCL blocks and PEG blocks, respectively, indicating the crystalline nature of both. These peaks were also visible in the hydrogel and the NP-loaded PECE with a slight decrease in intensity. This observation suggested that the crystalline structure of PCL is not affected, neither during the hydrogel formation nor upon the NP loading. Meanwhile, the PEG diffraction peak intensity weakens upon hydrogel formation and loading of the NPs, suggesting that the crystallisation ability of PEG blocks decrease upon hydration. As reported in other studies, the formation of micelles by PEG copolymers in water is hindered due to the excessively long molecular chains and structural characteristics [28,29], resulting in the formation of a hydrogel with a core-shell structure that reduces direct exposure of the IFN-loaded NPs to the medium, and prolongs IFN release, acting as a shield and ultimately creating a sustained release platform.

### 3.2. Evaluation of the Thermal Behaviour of the Hydrogels

**Figure 5:**
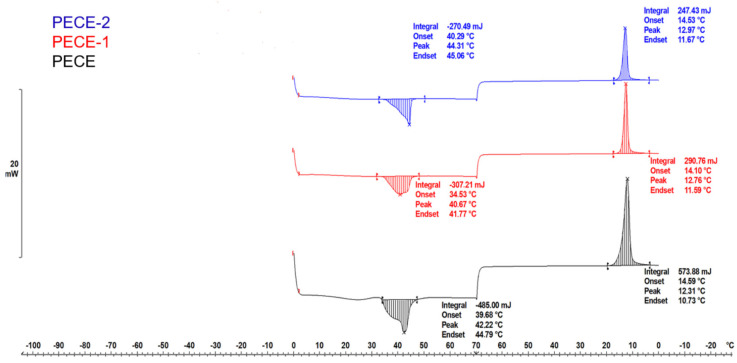
DSC thermograms of the PECE copolymer, PECE-1 and PECE-2.

**Figure 6:**
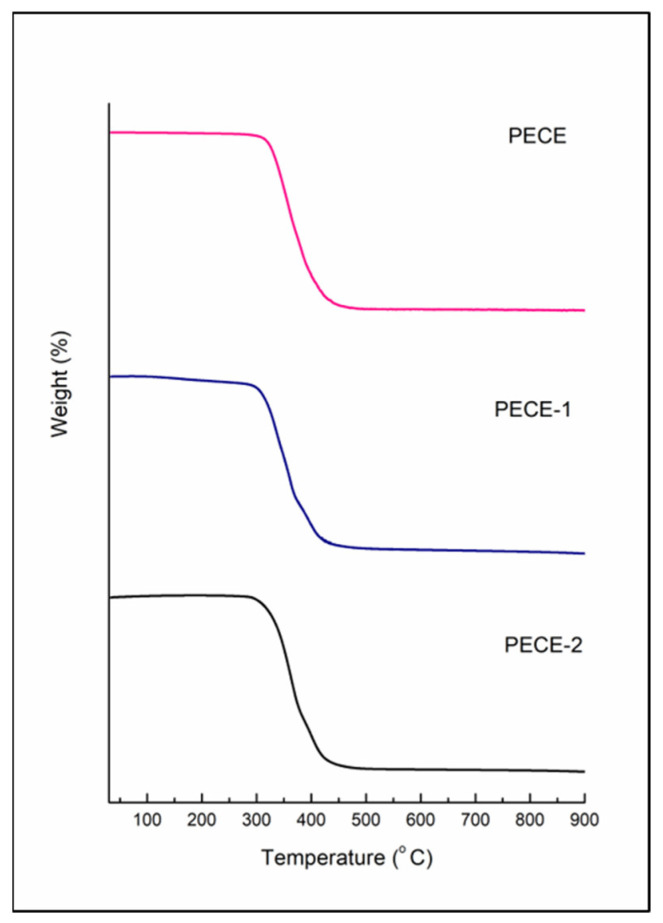
TGA thermograms of PECE, PECE-1 and PECE-2.

Differential scanning calorimetry (DSC) analysis was conducted to evaluate the transitions in the formulated hydrogels’ thermal behaviour compared to the native components. Figure 5 depicts the thermal curves of PECE copolymer, PECE-1 and PECE-2. Endothermic peaks were observed at 36 °C and 48 °C for the PECE copolymer. The two endothermic peaks correspond to the melting of PEG and PCL, respectively, in the dry state. In the hydrated state of PECE-1 and PECE-2, a single endothermic peak was observed at ~48 °C corresponding to the melting point of PCL. Endothermic peaks representative of the melting points of PEG and the NPs peaks were not evident due to their amorphous nature. The hydrophilic nature of PEG rendered it completely soluble in water at room temperature; hence no melting peak of PEG was evident in the hydrogel. Figure 5 highlights the cooling process of all the samples from 70 °C to 0 °C; single exothermic peaks were observed for all the samples. The peaks indicate the crystallisation of the samples occurring at ~18–20 °C increased in the hydrogel state and slightly decreased upon the addition of the NPs. The decrease in the crystallisation temperature upon addition of the NPs to the hydrogel is proposed to be due to small disruptions of the PECE chain. Similar results have been reported, where the addition of different concentrations of nano-hydroxyapatite powder to a PECE copolymer hydrogel resulted in a decrease in the crystallisation temperature [30]. Thermogravimetric analysis (Figure 6) was conducted to investigate the thermal stability of the PECE copolymer and hydrogels (PECE-1, PECE-2). The TGA thermogram demonstrates that the PECE copolymer commenced with decomposition at 290 °C, PECE-1 at 282 °C, and PECE-2 at 291 °C. The decomposition curve of all three demonstrated a major weight loss (95%), in the temperature range from 280 °C to 460 °C. The similarity in decomposition profiles signifies that the hydrogel preparation would not affect the chemical composition of the PECE copolymer, and that loading of NPs would not negatively affect the thermal stability of the hydrogel.

### 3.3. Particle Size and Distribution, Zeta Potential and Entrapment Efficiency of the Nanoparticles

Table 1 displays the particle size of the NPs and IFN-α2b loaded PBAE-NPs (IFNPs) to be 137.1 and 151 nm, respectively, demonstrating that the size increased with IFN-α2b loading and that the prepared NPs are within a good particle size range. Additionally, the PDI of the nanoparticles was reported to be 0.270 and 0.148 for the NP and IFNPs, respectively. The low PDI is an indication of the acceptably narrow size range distribution of the NPs. The zeta potentials were −23.9 and −16.4 mV for the NPs and IFNPs, respectively. Zeta potential analysis is a fundamental tool for assessing the surface charge properties of nanoparticles, which critically influence colloidal stability. Systems with high absolute zeta potential values (typically > ±20 mV) exhibit strong electrostatic repulsion, effectively preventing flocculation and aggregation. As such, zeta potential serves as a key indicator of formulation stability, ensuring long-term electrostatic stabilisation in drug delivery systems [31]. The zeta potential of both the drug-free and drug-loaded NPs was, thus, acceptable, although loading the NPs with IFN did lower the colloidal stability slightly. As shown in Table 1, the NPs achieved a high entrapment efficiency of 89% for IFN-α2b, confirming effective drug loading.

**Figure 7:**
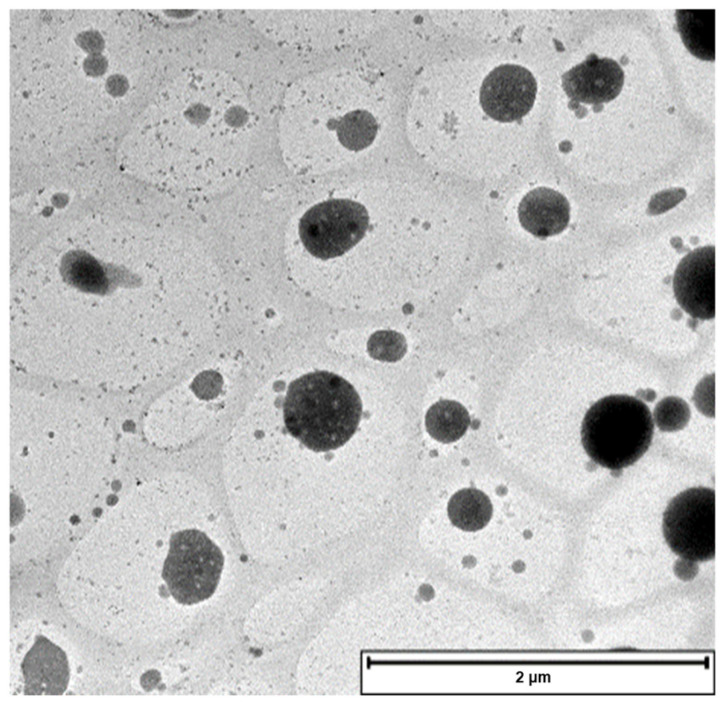
TEM image of PBAE drug-free nanoparticles. The scale bar is 2 µm.

The pH value of the media affected the size, PDI and zeta potential of the NPs, as demonstrated for the drug-free NPs. As evident in Table 2, the size changed in response to the acidic environment. As the pH value was adjusted to a more acidic environment from pH 7.4 to a slightly acidic tumour pH of 6.6 the size increased from ~180 to 216 nm with a narrow size distribution, while the zeta potential became more positive, from −11.4 mV at physiological pH to −6 mV at pH 6.6. The increase in size suggested the presence of a stable expanded core–shell structure that underwent protonation in the presence of an acidic environment. Under acidic conditions, PBAE NPs exhibited a pH-dependent reduction in zeta potential due to protonation of tertiary amines (–NR_2_ → –NR_2_H^+^), which neutralises the negative surface charge of PBAE and destabilises the nanoparticles. This charge neutralisation that occurred as the pH became more acidic (from −11 mV at pH 7.4 to −6 mV at pH 6.6) confirms the pH-responsive behaviour critical for tumour microenvironment-triggered drug release. The TEM image (Figure 7) confirmed the successful formulation of spherical, monodisperse nanoparticles (~100 nm) with smooth surfaces and no aggregation, which correlated with DLS hydrodynamic sizes (Table 1). The DLS data (Table 1 and Table 2) demonstrates a monomodal size distribution, confirming the overall consistency of the nanoparticle population. The decrease in size in the microscopic analyses was attributed to differences in the measuring environment, whereby TEM measurements were in the dry state and DLS in the hydrated state. Furthermore, the intact NP structures observed suggest successful formulation stability under the preparation conditions.

### 3.4. Assessment of Surface Morphology of the Hydrogels

**Figure 8:**
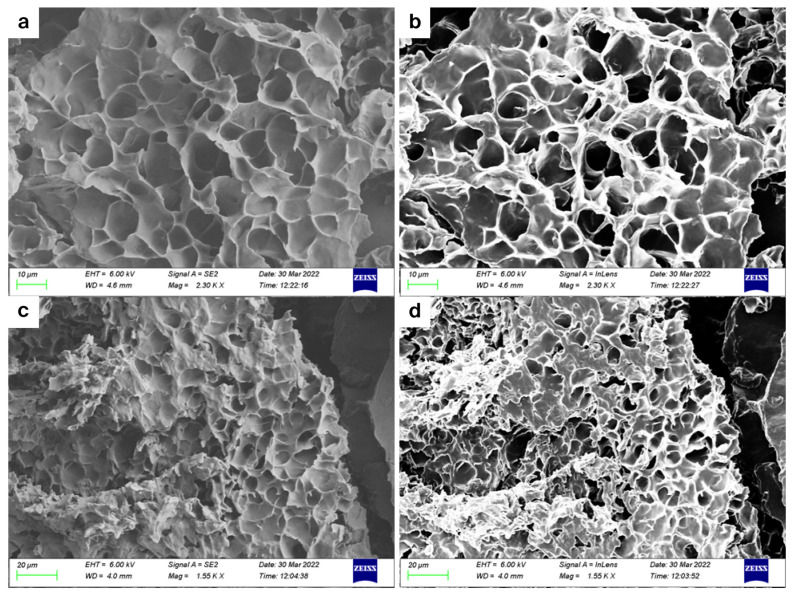
SEM images of PECE-1 (**a**) and (**b**) at 10 µm; PECE-2 (**c**) and (**d**) at 20 µm.

The SEM images of the PECE hydrogel depicted in Figure 8 indicate the presence of a porous structure, which acted as a matrix for the NPs. SEM has been the gold standard in the evaluation of surface topography. Nevertheless, the comparison between data is difficult to achieve due to the qualitative nature of the results [32]. The porosity analysis discussed hereunder supported the SEM results, further confirming the matrix-like structure of the hydrogel and the IFNPH.

### 3.5. Assessment of the Porosity Profile of the Hydrogels and NP-Loaded Hydrogels

The hydrogels’ porositometric analysis indicated differences in surface area, pore volume, and pore size when generated with varied concentrations of PECE and constant NP loading. A degree of porosity is vital for the diffusion of the NPs from the hydrogel, and hence drug release. Table 3 displays the results of porosimetric examination on hydrogels containing two concentrations of PECE: 20% *w*/*v* and 25% *w*/*v*, as well as hydrogel samples loaded with PBAE NPs. The results highlighted that PECE-1 exhibited a larger pore size and volume, but a smaller surface area compared to PECE-2. The presence of the NPs within PECE-2 decreased the pore size and increased the total surface area. The pore size and pore volume contributed to the cumulative surface area on the hydrogel surface pores, whereby the increase in pore diameter and depth increases the pore surface area, while decreasing the BET total surface area [33]. However, the increase in PECE concentration to 25% *w*/*v* for the PECE-3 resulted in a decline in pore size with a high BET surface area. PECE-4 exhibited a higher pore size, and a smaller surface area as compared to the PECE-3 hydrogel. The lower BET surface area of the PECE-4 compared to that of the PECE-3, despite the formation of pores, may further suggest that the pore formation occurs only on the surface of the hydrogels and not throughout the entire hydrogel.

In the context of physisorption, pores present in materials can be categorised according to their size into macro- (>50 nm), meso- (2–50 nm) and micropores- (<2 nm). The adsorption isotherms provide insightful information regarding pore size distribution [34,35]. According to this classification, the pore size distribution for 25% hydrogels (PECE-3 and PECE-4) lies within the macropore range, while the 20% hydrogels (PECE-1 and PECE-2) lie within the mesoporous range (Table 3). The PECE-3 and PECE-4 hydrogels exhibited a Type II isotherm with a H3 hysteresis loop indicating a monolayer-multilayer adsorption of a macroporous material [35]. The PECE-1 and PECE-2 hydrogels exhibited a Type III isotherm with a H3 hysteresis loop suggestive of the presence of a multilayer material with complete pore filling including capillary condensation that occurs in the presence of mesopores [34,35]. The presence of an H3 hysteresis loop indicates the presence of an interconnected pore system of specialised distribution within the hydrogel matrix [34]. Similar results were visualised on the SEM images. The interconnection of pores is vital for the diffusion of liquid within the hydrogel allowing the diffusion of the NPs out of the hydrogel, and subsequent drug release.

### 3.6. Assessment of Rheological Properties of the Hydrogel

**Figure 9:**
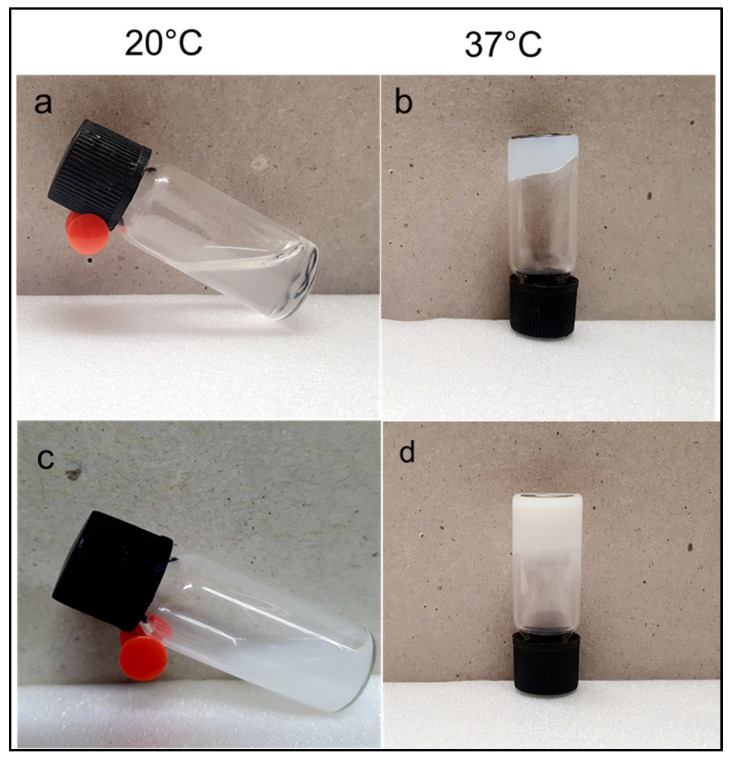
Photographic images of the sol–gel transition of hydrogels at room and body temperature (**a**) solution of PECE-1; (**b**) gel of PECE-1 and (**c**) solution of PECE-2; (**d**) gel of PECE-2.

**Figure 10:**
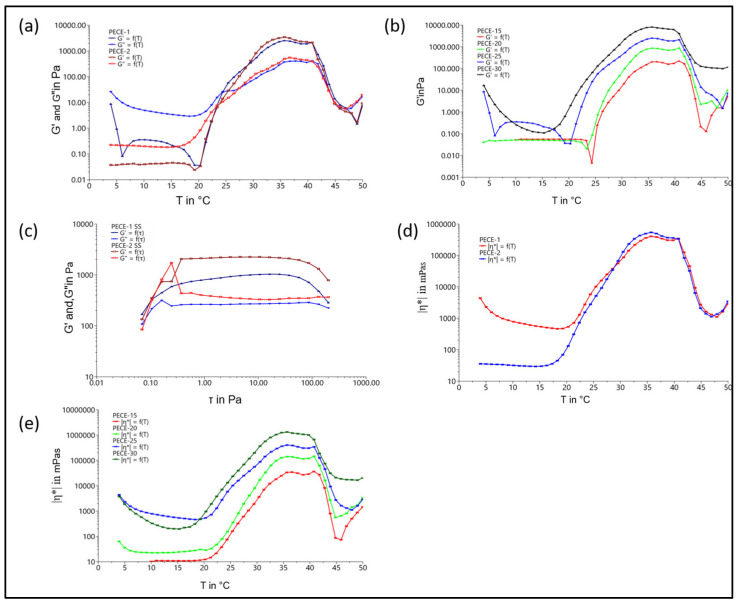
(**a**) Temperature ramp of PECE-1 and PECE- 2; (**b**) temperature ramp of PECE hydrogel at different concentrations (15%, 20%, 25% and 30% *w*/*v*); (**c**) stress sweep of PECE-1 and PECE-2 at 25 °C; (**d**) viscosity of PECE-1 and PECE-2; and (**e**) viscosity of PECE hydrogel.

The sol–gel transition of PECE hydrogels at different concentrations was studied to confirm the suitability of the system for application at body temperature. Figure 9 visually presents the temperature-dependent sol–gel transition of the PECE hydrogel loaded with PBAE NPs at a hydrogel concentration of 20% *w*/*v* using the test-tube inversion method. The sol–gel transition of the PECE was concentration-dependent, below which the transition will not occur; this is referred to as the critical gelling concentration (CGC), which in this case is 15% *w*/*v*. The sol–gel transition of 20% *w*/*v* PECE was transient with a window, i.e., between 26 °C and 44 °C. Further increasing the concentration broadened the sol–gel transition window with increasing temperature. As evident in Figure 10b, the window shifted from 26 °C to 24 °C and from 44 °C to 47 °C at 20–25% *w*/*v*. The loading of the NPs into the hydrogel resulted in a slight decrease in the transition temperature from 26 °C to 25 °C. The 20% *w*/*v* PECE possessed a comparatively favourable sol–gel transition. This concentration was selected as the preferred concentration based on the ease of preparation, ease of administration, and minimal discomfort upon the administration, which is discussed in further detail in the following section. Thus, the in-depth physicochemical characterisations of PECE-1 and PECE-2 are presented herein.

Moreover, the storage modulus (G′) and the loss modulus (G″) signifying the elastic and viscous behaviour, respectively, were monitored as a function of temperature. In Figure 10a,b, the G″ was higher than G′ at low temperature, corresponding to the sol state. As the temperature increases a crossover occurs around 24 °C from sol state to gel state (lower transition), at which the G′ was equal to the G″. At the second crossover above 42 °C (upper transition), the PECE transitioned from gel state to sol state.

The viscosity increased as the temperature increased, depicted in Figure 10d, further confirming transition from sol state into gel state. Additionally, the viscosity demonstrated a concentration-dependent manner, as evident in Figure 10e. The oscillatory stress sweep measurement on PECE-1 and PECE-2 further confirmed the hydrogel formation. The hydrogels, within their linear viscoelastic region approaching 100 Pa in terms of shear stress, demonstrated that the elastic modulus G′ dominated the viscous modulus G″ with a magnitude of 210 Pa, noted in Figure 10c.

### 3.7. Assessment of the Syringeability of the Hydrogels

**Figure 11:**
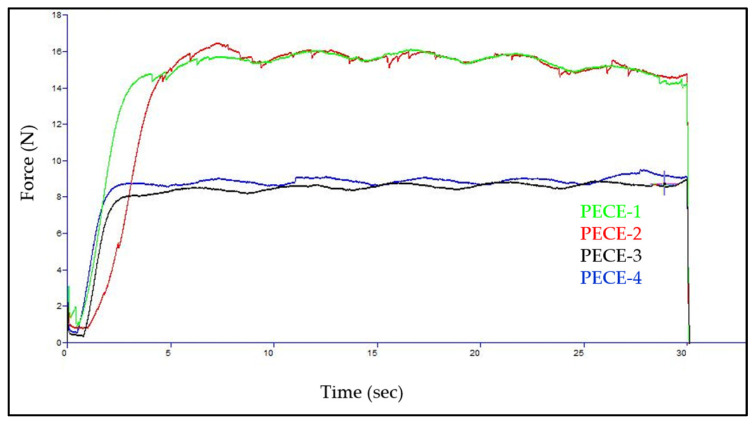
Required force to inject PECE hydrogel at different concentration from 1 mL syringes equipped with 27 G needle, PECE-1 (20% *w*/*v* hydrogel), PECE-2 (20% *w*/*v* NP-loaded hydrogel), PECE-3 (25% *w*/*v* hydrogel), and PECE-4 (25% *w*/*v* NP-loaded hydrogel).

Texture analysis was applied to establish the maximum force required to inject hydrogels, and the findings are presented in Figure 11. The hydrogels and NP-loaded hydrogel syringeability experiments were carried out using 27-gauge needles. The texture analyser force required to inject the PECE hydrogels through a 1 mL syringe connected to 27G needles was employed to assess syringeability. The injection force includes three elements: the force required to overcome syringe plunger resistance, delivering kinetic energy to the liquid under evaluation, and propelling the liquid through the needle [36]. PECE hydrogels (20 and 25% *w*/*v*) required 8 N and 16.1 N, respectively, to inject them through 27G needles, whereas nano-dispersed PECE hydrogels (20 and 25% *w*/*v*) required 9 N and 16.5 N, respectively. The concentration of the hydrogel and the dispersion of NPs in the hydrogel were two factors that influenced the injection force, resulting in an increased maximum force required for injection as these factors increased. These results are within two-thirds of the maximum force recommended for a manual injection, which is 30 N. As a result, the nano-dispersed hydrogel PECE is appropriate for injection via a variety of routes of administration.

### 3.8. In Vitro Release Behaviour of IFN-α2b from IFNPs and IFNPH

**Figure 12:**
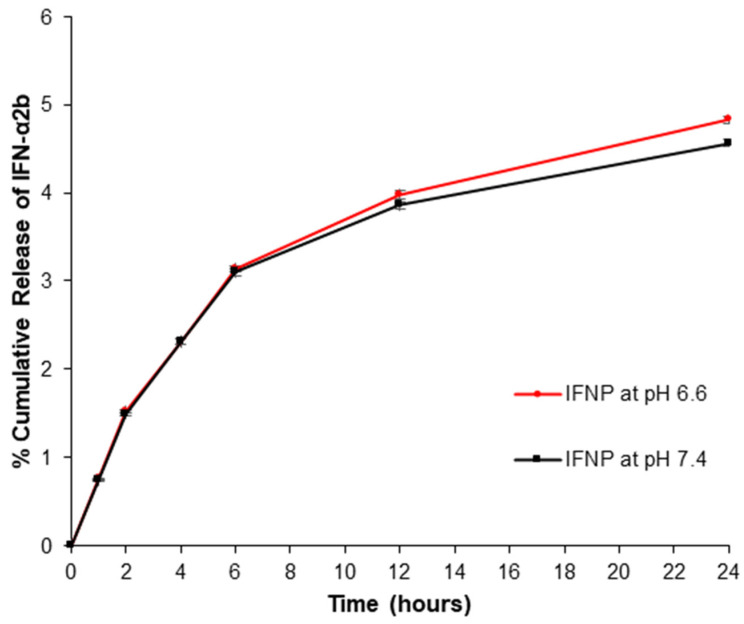
In vitro release profiles of immunoenzymatically detected IFN-α2b from IFNP at 37 °C at different pH values.

**Figure 13:**
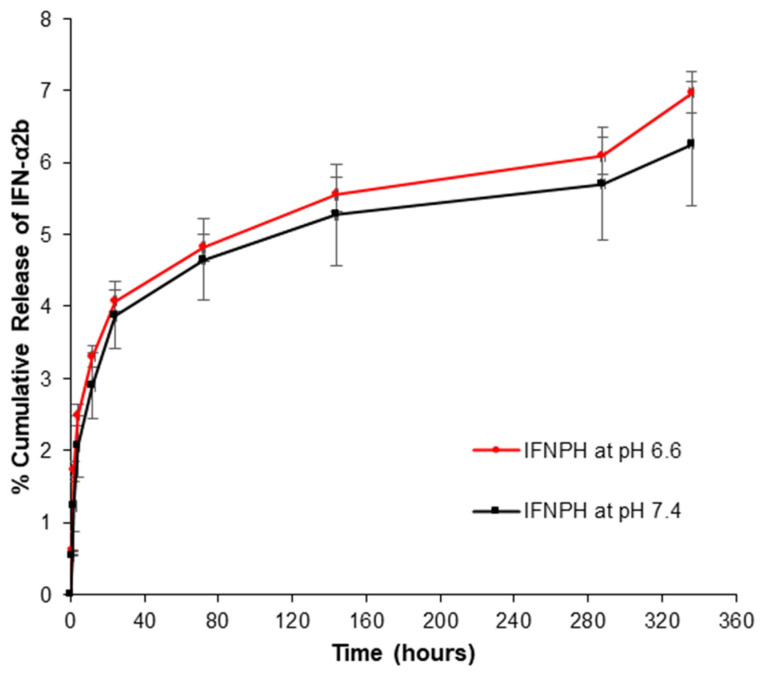
In vitro release profiles of immunoenzymatically detected IFN-α2b from IFNPH at 37 °C at different pH values.

The in vitro release of IFN-α2b from the IFNP was investigated over 24 h, and over 2 weeks from the IFNPH in PBS at different pH values (7.4 and 6.6, at 37 °C). PBS (pH 6.6) was used to simulate pH conditions relevant to the extracellular tumour tissues, whereas PBS pH 7.4 was used to simulate the normal extracellular cells conditions. Figure 12 and Figure 13 depict the release patterns of IFN-α2b, and the influence of the pH on the drug release. The release from the IFNPH revealed a biphasic release pattern, where an initial burst release was followed by sustained release. The cumulative percentage release from the IFNPH was 3.8% and 4.1% within the first 24 h at pH 7.4 and 6.6, respectively. The influence of the pH on the drug released from the IFNPH revealed that the release was higher at the pH of the tumour extracellular microenvironment. This was further explained by the behaviour of PBAE forming the NPs, which possess pH-sensitive amine groups; at pH 6.6 there was the ionisation of the PBAE-NPs. This resulted in swelling and an increase in solubility of the nanoparticle matrix, enabling the increased release of the entrapped IFN at the tumour microenvironment pH, while at the same time protecting the entrapped IFN from the more acidic environment, and providing controlled release. The cumulative percentage release from the IFNPH was 6.3% and 7.0% after 14 days at pH 7.4 and 6.6, respectively.

The cumulative percentage release from the IFNPs was 4.5% and 4.8% following 24 h at pH 7.4 and 6.6, respectively, highlighting the slight, though evident pH-responsiveness of the nanoparticulate system. The initial burst release of IFNPs was greater compared to that of IFNPH due to the release of surface-located IFN-α2b, which was accessible for immediate release. The controlled release of IFN-α2b from the IFNPH formulation is beneficial as it prevents chronic activation of the type I IFN pathway. Evidence has reported that chronic exposure to IFN may result in the development of resistance [3,37].

Student’s paired *t*-test was used to compare the release from the IFNP after 24 h at the two different pH values; there was no significant difference between the groups, although the rate of IFN-α2b release at the acidic pH was slightly higher (p=0.06). For the release from the IFNPH after 14 days at both pH values, there was a significant difference between the groups (*p* = 0.0006), with higher levels of IFN-α2b released at the pH of the tumour microenvironment. A comparison between the release profile from the IFNP vs. the IFNPH after 24 h indicating that the hydrogel significantly controlled the release of IFN at pH 7.4 (*p* = 0.021), highlighting the pertinence of the hydrogel in further reducing off-target IFN effects.

Feczkó et al. (2016) reported the release of IFN-α from PLGA nanoparticles, and the formulations displayed sustained release characteristics with a base line release after one hour of 20–25 ng/L [10]. In this investigation, the IFN-α release from the IFNPH was 90.3 ng/L and 101.3 ng/L after one hour at pH 7.4 and pH 6.6, respectively. The pH-dependent IFN release (Figure 12 and Figure 13) arises from protonation-induced PBAE destabilisation and ester bond hydrolysis in a more acidic environment. This resulted in a swollen core-shell structure, which facilitated IFN release in a simulated tumour microenvironment. This system, thus, enables pH-responsive, controlled and site-specific tumoral drug delivery (reduced dosing frequency), and while release at physiological pH was lower than at tumoral pH, potential premature release at neutral pH or off-target effects in inflamed tissues may warrant further formulation optimisation (e.g., pK_a_ tuning) to further minimise release at pH 7.4 and mitigate potential off-target release at neutral pH for improved clinical translation. The system was proposed to release IFN-α over 2 weeks (14 days), but it is evident from the results that controlled release could be over an extended period, thus reducing the need for multiple applications.

### 3.9. Degradation Analysis of the Formulations

**Figure 14:**
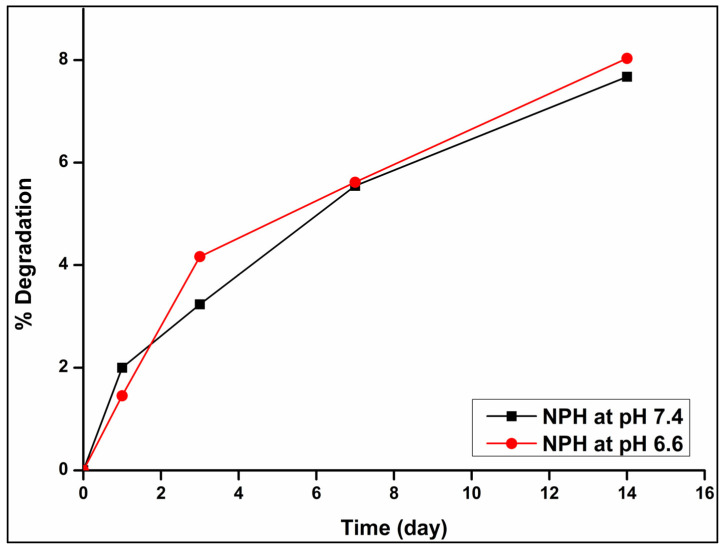
Biodegradation behaviour of the NPH in PBS solution (pH 6.6 and 7.4) at 37 °C. (The error bars were small, and the standard deviation ranged between 0 and 4.62 × 10^−15^).

Biodegradable hydrogels are of pertinence in ocular disease as they would degrade slowly, negating the need for frequent administration. The nano-dispersed PECE hydrogel NPH showed minimal degradation with 8% and 7.67% degradation on the 14th day at pH 6.6 and 7.4, respectively. The difference between the degree of degradation at pH 6.6 and 7.4 was not significant (*p* = 0.5536); however, the degradation rate was slightly higher at pH 6.6 simulating the tumour microenvironment, correlating with the release behaviour observed from the IFNPH. This slow degradation could facilitate a decreased frequency of administration. The degraded formulation did not exhibit a colour change, simultaneously the formulation displayed low water uptake indicating that upon subconjunctival administration, the hydrogel will not swell extensively, limiting tissue damage. As evident in Figure 14 the slow erosion of the hydrogel confirms that further release of the IFN-α2b from the IFNPH is possible as only 8% of the hydrogel has eroded after 14 days.

### 3.10. Biocompatibility Evaluation of the Formulations

**Figure 15:**
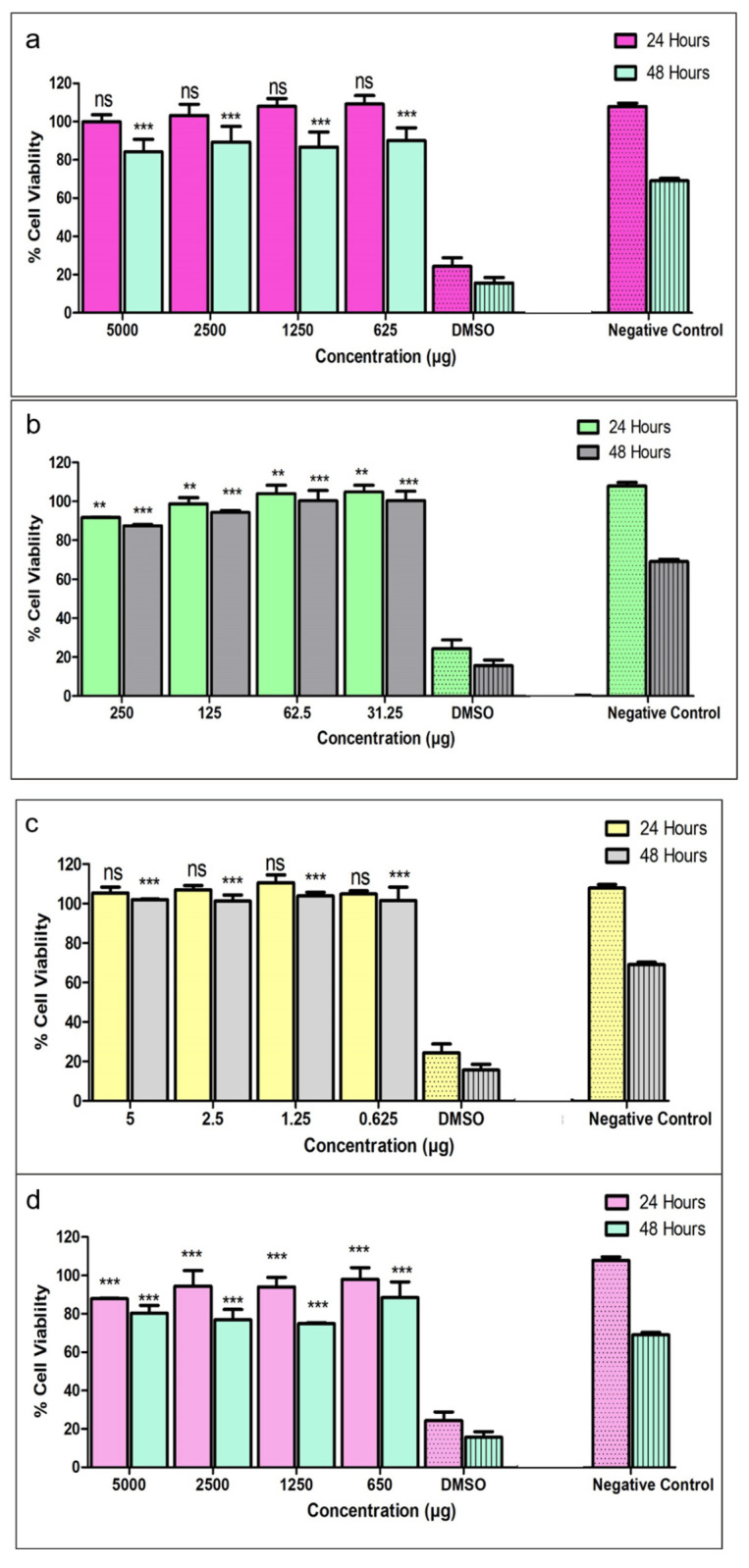
MTT assay of viability of HRPE cells following 24 and 48 h treatment with (**a**) PECE hydrogel; (**b**) PBAE-NP; (**c**) IFN-α2b; and (**d**) IFNPH at different concentrations (Data represents n = 3, mean ± SD). ** Indicates *p* < 0.01, *** indicates *p* < 0.001, and (ns) indicates no significant difference when compared to negative control. 5% ^v^/_v_ DMSO and culture medium were used as positive and negative controls, respectively, indicated by the patterned columns.

An MTT assay was used to evaluate the cytotoxicity of the formulations on a HRPE cell line. The assay was employed to measure viability and cyto-compatibility with HRPE after 24 and 48 h treatment with PECE hydrogel, NPs, IFN-α2b, and the IFNPH at different concentrations. The results showed that the formulations displayed acceptable levels of cell viability, with minimal cytotoxicity effects on HRPE after 24 and 48 h, and that the viability was dose-dependent as evident in Figure 15. Although the viability after 48 h was reduced for all systems evaluated, the percentage cell viability was greater than 70% [38], therefore the NPs, hydrogel, and final IFNPH can be regarded as non-cytotoxic; indicating that the IFNPH is non-cytotoxic to HRPE cell lines. The cells’ metabolic waste products may have accumulated after 48 h of treatment, resulting in increased cytotoxicity evident in the MTT assay results [39]. Student’s unpaired *t*-test was used to compare the cytotoxicity at 24 h vs. 48 h. There was no statistical significance between the cytotoxicity after 24 h treatment (*p* = 0.4727) when comparing the PECE hydrogel concentrations from 5000 to 625 µg/mL with the negative control at 24 h. The results after 48 h treatment compared to the negative control showed a significant difference (*p* < 0.001). Comparison of the cytotoxicity of the PBAE-NP with the negative control was not significant and significant (*p* < 0.01 and *p* < 0.001) after 24 h and 48 h, respectively. Comparison of cytotoxicity of the IFN-α2b with the negative control showed no significant difference at 24 h and a significant difference (*p* < 0.001) after 48 h. The IFNPH showed a significant difference (*p* < 0.001) after 24 h and 48 h when compared to the negative control. The increased toxicity after 48 h might be a stress response to accumulation of toxic elements in the growth medium [40].

### 3.11. Assessment of the Stability and Bioactivity of the Released IFN-α2b from the IFNPH

**Figure 16:**
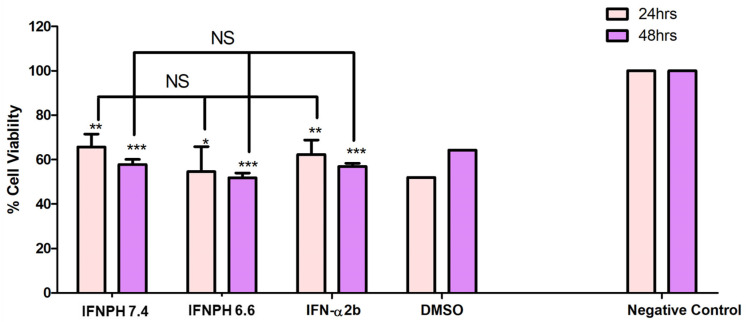
In vitro cytotoxicity activity of IFN-α2b released from the IFNPH formulation on A172 cells at different pH values. 5% *v*/*v* DMSO and culture medium were used as positive control and as negative control, respectively (Data represent n = 3, mean ± SD). * Indicates *p* < 0.05, ** indicates *p* < 0.01, *** indicates *p* < 0.001 when compared to negative control. NS indicates no significant difference when compared to standard IFN-α2b.

The biological activity of the released IFN-α2b was investigated as shown in Figure 16. The results show that the biological activity of the released IFN-α2b was maintained for the duration of the study. The structural integrity of IFN-α2b was maintained during the preparation and release from the IFNPH. A student’s unpaired *t*-test was used to compare the released IFN-α2b at the different pH values with standard IFN-α2b. There was no significant difference after 24 and 48 h treatment. When compared to the negative control there was a significant difference after 24 h at pH 7.4 (*p* < 0.05) as well as at pH 6.6 (*p* < 0.01). After 48 h there was a significant difference at pH 7.4 and pH 6.6 (*p* < 0.001), indicating that the released IFN-α2b was active and is cytotoxic to the A172 cells. The cytotoxic effect on the A172 cancer cells was more notable at pH 6.6 highlighting that a slightly higher level of biologically active IFN was available to exert its effect at the tumour microenvironment pH.

## 4. Discussion

Interferons are glycosylated polypeptides produced in the body with antineoplastic, antiviral, and antibacterial properties [41]. The antitumour effect is achieved through a combination of antiangiogenic, antiproliferative, cytotoxic effects, as well as cytostatic effects and enhancement of cell lysis [41]. IFN-α2b has been used in management of different types of malignancies. The clinical efficiency of IFN-α2b is hindered due to the high sensitivity of IFN-α2b to degradation and clearance by the reticuloendothelial system [10]. Thus, this work presents the formulation of a thermo-responsive hydrogel loaded with pH-responsive NPs as a platform for site-specific delivery of IFN-α2b for ocular surface tumour therapy.

The PECE copolymer was synthesised successfully by ring opening polymerisation, and the FTIR spectra revealed the presence of the characteristic peaks of PECE copolymer [18,22]. The findings correlate to previous reports, and the ^1^H-NMR confirmed the synthesis of the PECE copolymer [42,43].

The PECE hydrogel possessed thermo-responsive and biodegradable properties, which have been reported previously, as well as functionality as in situ-forming depot for a sustained drug delivery system [22,44,45]. In this study, the sol–gel transition of the PECE copolymer was investigated employing the test tube-inversion method and rheological studies [46]. The amphiphilic nature of the PECE copolymer is owed to its hydrophilic PEG block and hydrophobic PCL block. The sol–gel transition is mainly due to micelle aggregation; the PECE copolymer tends to form micelles with a hydrophobic PCL core and a hydrophilic PEG shell, whereas the gel-sol transition is due to acceleration of the molecular motion of PCL resulting in the breakage of the micelle structure; the mechanism of hydrogel formation for PECE copolymer has been previously reported [47].

The rheological parameters such as viscosity, loss modulus and storage modulus are important parameters to consider when administering the hydrogel through injection. The moduli measure the elasticity or rigidity of the hydrogel upon shear (i.e., injection), while the viscosity measures the ability of the material to resist deformation upon stress, during hydrogel formulation injection [48,49]. The results indicated that higher PECE concentrations result in higher G′ values and viscosity, as a result, the maximum force necessary for injection was increased. Lower concentrations provide better injectable capabilities, but lower hydrogel strength, highlighting that the final copolymer solution concentration must balance the effects of numerous parameters. To minimise the discomfort and eye damage, 30 N is the recommended maximum force which means the lower the force, the better the treatment process [50]. In this study, 20% *w*/*v* PECE was selected as the preferred concentration based on the ease of preparation and ease of administration, which is proposed to result in minimal discomfort to the patient.

The NPs synthesised were in the nano size range with excellent polydispersity (low PDI) and zeta potential. The average particle size and PDI are essential physical properties to consider as they affect the encapsulation efficiency, stability, biodistribution, drug release profile and cellular uptake [51,52,53]. The DLS studies show that the prepared IFNPs had an average size of 151 nm with an encapsulation efficiency of 89% with a narrow PDI. Furthermore, the TEM images generated revealed that the formulated NPs are uniform and spherical shape with an intact structure within the nano size range and verified the DLS results. The pH-sensitive PBAE-NPs were developed and loaded into the PECE hydrogel to enable site-specific delivery of IFN-α2b to ocular surface tumour cells.

IFN-α2b was released at a slightly higher rate from PBAE-NPs in the acidic environment simulating the extracellular tumour microenvironment. The inclusion of core-shell nanoparticles increased the loading capacity of the drug and provided pH-responsiveness to the acidic tumour microenvironment, as well as protecting the entrapped drug. As a result, the concentration gradient is reduced and the release pathway extended, furthermore, shielding the entrapped IFN-α2b against rapid clearance, and degradation at lower pH of the tumour microenvironment. Thus, the pH-responsive NPs were stable with drug release occurring at a controlled yet slightly higher level at the pH of the tumour microenvironment vs. physiological pH, indicating that this drug delivery system can reduce unwanted systemic side effects of anticancer drugs during cancer therapy by providing pH-sensitive drug release [54]. The PBAE-NPs would proposedly be stable following diffusion from the hydrogel matrix into the solid tumour microenvironment and degrade in the extracellular and late endosomes/lysosomes where the pH is approximately 5–6 and 4–5, respectively, rendering them suitable for extracellular and cytoplasmic drug delivery [25].

Du Toit et al. (2021) [55] designed an injectable nano-enabled thermogel of PECE for controlled delivery of p11 anti-angiogenic peptide for the potential treatment of ocular angiogenic disorder. The release of the peptide from the nano-thermogel system was proposed to be due to diffusion mediated release through the gel matrix, with minimal swelling and ultimate erosion of the hydrogel [55]. The release of IFN-α2b from the IFNPH was proposed to follow a similar release pattern. When evaluating the release kinetics of proteins from nanosystems dispersed into hydrogels, it is essential to consider that with hydrogel systems, 100% protein release is hardly achieved. The protein might get physically entrapped in the highly entangled hydrogel network, limiting the diffusivity. The release kinetics proposed that the size of the protein plays the primary role in its diffusivity, in addition to the density of the hydrogel [56]. Fador-Kardos et al. (2020) [12] reported the effect of pH on the release profile of IFN-β, the protein was loaded into nanoparticles with high encapsulation efficiency (>95%) for the management of multiple sclerosis. The in vitro release of IFN-β revealed a decline in the release profile by the end of the two-week study; the proposed explanation was that the pH caused aggregation of the protein [12]. The limited release behaviour reported previously for IFNP at both pH values was attributed to inactivation by long-term exposure to physiological temperature and acidification of the medium. Determining IFN-α2b levels via ELISA provides an indicator of its stability [57], which was further confirmed by assessing the cytotoxic activity on the A172 glioblastoma cells. In vitro experiments in this investigation demonstrated the stability and bioactivity of the released IFN-α2b from IFNPH for the duration of the study, specifically at the lower pH of the tumour microenvironment; thus, the IFNPH exhibits protective properties over the incorporated bioactive.

Safety assessment of the formulations in vitro revealed that at concentrations ranging from 5000 to 0.625 µg/mL employed in the study, were within acceptable levels of cell viability, with no significant cytotoxicity effects on HRPE cells. Similar results were reported in other studies, confirming the biocompatibility of the PECE hydrogel [54]. Furthermore, concentration influences the percentage of cell viability, as higher concentrations are associated with lower viability. The difference in cell viability was statistically significant between the varied concentrations at the two different time points (*p* < 0.001) in all the formulations. The increased toxicity was purportedly due to a stress response to toxic elements in the growth medium and metabolic build-up.

## 5. Conclusions

In this work, PBAE-NPs and PECE copolymer were successfully synthesised. The triblock copolymer PECE was loaded with PBAE-NPs to prepare a nano-dispersed pH- and thermo-responsive hydrogel formulation. Loading the IFN-α2b in a hydrogel isolates the drug from the releasing medium; the inclusion of core-shell nanoparticles increases the loading capacity of the drug and provided pH-responsiveness to the acidic tumour microenvironment, releasing the entrapped drug at higher levels within the tumour microenvironment compared to normal physiological conditions, while also, shielding the IFN-α2b against rapid clearance, and degradation at lower pH of the tumour microenvironment.

Rheological measurements showed that the gelation points of the PECE hydrogel composites were adequate for subconjunctival injection. The gelation temperature could be adjusted by adding PBAE-NPs into the PECE matrix. The pH-protectiveness of the IFNPH hydrogel composites demonstrated that they could be employed for protecting the IFN from high levels of degradation and providing controlled release of IFN at tumour sites. Therefore, the IFNPH hydrogel nanocomposite is a prospective candidate as an injectable biomaterial for ocular drug delivery to tumour sites. The system demonstrated controlled release of IFN-α exceeding the 2-week (14 day) investigation period, thus reducing the need for multiple applications. The bioactivity of IFN-α released from the IFNPH was successfully demonstrated in the presence of an ocular tumour cell line, highlighting its potential application in the treatment of ocular surface tumours.

Further investigation of the duration of release and IFN levels in vitro and in vivo are future considerations. Although the concept of injectable and pH thermo-responsive hydrogel nanocomposites has been introduced in this study, further research is required to validate the application of such systems in tumour delivery via preclinical investigation in an appropriate animal model.

## Figures and Tables

**Table 1:** Particle size, PDI and zeta potential of unloaded and loaded nanoparticles and drug entrapment efficacy of loaded nanoparticles.

Sample	Size (nm)	PDI (a.u)	Zeta Potential (mV)	% EE
NP	137.1 ± 0.47	0.270 ± 0.002	−23.9 ± 0.58	-
IFNP	151 ± 1.46	0.148 ± 0.03	−16.4 ± 0.25	89 ± 0.35

Note: Values are expressed as (*n* = 3, mean ± SD).

**Table 2:** Effect of pH of the medium on the size, PDI and zeta potential of the drug free NPs.

Formulation/Media	Size (nm)	PDI (a.u)	Zeta Potential (mV)
NP/H_2_O	137.1 ± 0.47	0.270 ± 0.002	−23.9 ± 0.58
NP/PBS 7.4	179.9 ± 1.72	0.115 ± 0.12	−11.4 ± 0.61
NP/PBS 6.6	216.9 ± 4.70	0.079 ± 0.009	−6.12 ± 1.05
NP/PBS 5.5	230.6 ± 5.62	0.137 ± 0.018	−3.36 ± 0.67

Note: Values are expressed as *n* = 3, mean ± SD.

**Table 3:** Porositometric analysis of lyophilised PECE hydrogels and nanoparticulate-loaded PECE hydrogels.

Porositometric Parameters	PECE-1	PECE-2	PECE-3	PECE-4
Surface Area				
Single point surface area (m^2^/g)	0.1637	0.2112	0.2330	0.2085
BET Surface Area (m^2^/g)	0.1462	0.2088	0.4479	0.2398
BJH Adsorption cumulative surface area of pores between 17.000 Å and 3000.000 Å diameter (m^2^/g)	0.032	0.084	0.272	0.355
BJH Desorption cumulative surface area of pores between 17.000 Å and 3000.000 Å diameter (m^2^/g)		0.1172	0.2823	
Pore Volume				
Single point adsorption total pore volume of pores (cm^3^/g)	0.001478	0.00032	0.00075	0.003647
BJH Adsorption cumulative volume of pores between 17.000 Å and 3000.000 Å diameter (cm^3^/g)	0.002437	0.00222	0.00126	0.003468
BJH Desorption cumulative volume of pores between 17.000 Å and 3000.000 Å diameter (cm^3^/g)	0.002500	0.00217	0.00125	
Pore Size				
Adsorption average pore width (4V/A by) (Å)	1916.746	61.5310	67.1867	608.3260
BJH Adsorption average pore diameter (4V/A) (Å)	98.191	1054.71	185.162	390.541
BJH Desorption average pore diameter (4V/A) (Å)	47.168	742.370	177.708	

Note: PECE-1 (20% *w*/*v* hydrogel), PECE-2 (NP loaded into 20% *w*/*v* hydrogel), PECE-3 (25% *w*/*v* hydrogel) and PECE-4 (NPs loaded into 25% *w*/*v* hydrogel).

## Data Availability

All data generated or analysed during the current study are available from the corresponding author upon reasonable request.

## Abbreviations

The following abbreviations are used in this manuscript:

3AP	3-Amino-1-propanol
ANOVA	Analysis of variance
BJH	Barrett, Joyner and Halenda model
BDDA	1,4-Butanediol diacrylate
BET	Brunauer–Emmett–Teller
CGC	Critical gelling concentration
CDCl_3_	Deuterated chloroform
DSC	Differential scanning calorimetry
DOX	Doxorubicin
DMEM F12	Dulbecco’s modified Eagle medium mixture with Ham’s
DLS	Dynamic light scattering
EE	Encapsulation efficiency
EPR	Enhanced permeation and retention
ELISA	Enzyme-linked immunosorbent assay kit
Ɛ-CL	Ɛ-caprolactone
FBS	Foetal bovine serum albumin
FTIR	Fourier transform infrared
HMDI	1,6-hexamethylene diisocyanate
^1^H-NMR	**^1^**H-nuclear magnetic resonance
HRPE	Human retinal pigment epithelial cells
IFN-α2b	Interferon-alpha2b
IFNPs	Interferon-alpha2b loaded PBAE-NPs
IFNPH	Interferon-alpha2b loaded PBAE-NPs/PECE hydrogel
IFN-β	Interferon beta
(G″)	Loss modulus
MTT	3-(4, 5-dimethylthiazol-2-yl)-2,5-diphenyl tetrazolium bromide
NPs	Nanoparticles
PECE	Poly (ethylene glycol)-poly (caprolactone)-poly (ethylene glycol)
PEG-PCL-PEGE	Poly (ethylene glycol)-poly (caprolactone)-poly (ethylene glycol)
PECE-1	20% *w*/*v* PECE hydrogel
PECE-2	NPs dispersed into 20% *w*/*v* PECE hydrogel
PECE-3	25% *w*/*v* PECE hydrogel
PECE-4	NPs dispersed into 25% *w*/*v* PECE hydrogel
PEGIFN-α2b	Pegylated interferon
PBS	Phosphate buffer saline
PBAE	Poly (beta-amino ester)
PBAE-NPs	Poly (beta-amino ester) nanoparticles
PEG	Poly (ethelyene glycol)
PCL	Poly (caprolactone)
mPEG	Poly(ethylene glycol) methyl ether
mPEGA	Poly(ethylene glycol) methyl ether acrylate
PDI	Polydispersity index
RES	Reticuloendothelial system
SEM	Scanning electron microscopy
SD	Standard deviation
Sn(Oct)_2_	Stannous octoate
(G′)	Storage modulus
TGA	Thermogravimetric analysis
(W_NPs_ + W_PECE_)	Total mass of the solid component
TEM	Transmission Electron Microscopy
WNPs	Mass ratio of the NPs
XRPD	X-ray powder diffraction
